# Percutaneous ballon compression, a better choice for primary trigeminal neuralgia compared to microvascular decompression?

**DOI:** 10.3389/fsurg.2024.1517064

**Published:** 2025-01-07

**Authors:** Yuwei Shi, Wenhu Liu, Shaopeng Peng, Jianxiong Liu

**Affiliations:** ^1^ First Clinical Medical School, Gansu University of Chinese Medicine, Lanzhou, Gansu, China; ^2^ Department of Neurosurgical Intensive Care Unit, Gansu Provincial Hospital, Lanzhou, Gansu, China; ^3^ Department of Neurosurgery, Ward 2. Gansu Provincial Hospital, Lanzhou, Gansu, China

**Keywords:** primary trigeminal neuralgia (PTN), percutaneous balloon compression (PBC), trigeminal microvascular decompression (MVD), efficacy & safety, operation duration, postoperative hospital stay

## Abstract

**Objective:**

Demonstrate the superiority of percutaneous balloon compression (PBC) in the treatment of primary trigeminal neuralgia (PTN) compared to trigeminal microvascular decompression (MVD).

**Methods:**

Clinical data, including immediate, short-term, and long-term pain relief, complications, duration of the operation, and postoperative hospital stay, were retrospectively analyzed for 114 patients diagnosed with PTN who were treated with either PBC or MVD between January 2018 and December 2021.

**Results:**

There were no statistically significant differences observed in the pain relief rates between the two surgical methods at 24 h postoperatively (MVD: 91.07%, PBC: 96.55%), at 6 months postoperatively (MVD: 87.5%, PBC: 94.8%), at 1 year postoperatively (MVD: 83.90%, PBC: 94.80%), and at 2 years postoperatively (MVD: 78.60%, PBC: 72.40%). However, the incidence of meningitis following MVD was significantly higher than that following PBC (*P* < 0.005). Additionally, both the duration of the operation and the length of the postoperative hospital stay in the MVD group were longer than those in the PBC group (*P* < 0.005).

**Conclusion:**

PBC demonstrates efficacy comparable to MVD while offering a simpler procedure, improved safety, and a shorter postoperative hospital stay. Therefore, it may serve as a viable alternative to MVD and could become the preferred surgical approach for treating PTN in the future.

## 1 Introduction

Trigeminal neuralgia (TN) is a condition characterized by recurrent, unilateral pain in the distribution of the trigeminal nerve. The pain often described as severe, resembling an electric shock or stabbing sensation. Each episode typically lasts 1–2 min and can be triggered by minor stimuli such as speaking, chewing, brushing teeth, or touching certain areas of the face. The trigeminal nerve is responsible for sensation in the face and has three distinct branches: V1, V2, and V3. The V1 branch, also known as the ophthalmic division, innervates the forehead, upper eyelids, and the region surrounding the eyes. The V2 branch, or maxillary division, supplies sensation to the lower eyelids, cheeks, and upper lip. Finally, the V3 branch, called the mandibular division, provides sensory input to the lower lip, chin, and jaw, while also containing motor fibers that control mastication muscle (1). The pathological mechanisms underlying TN remain incompletely understood, and no single hypothesis adequately explains the clinical symptoms observed. Current perspectives suggest that genetic mutations, anatomical alterations, and neurophysiological factors are the primary contributors to the development of TN (2).

TN is classified into primary and secondary subtypes. Pharmacological therapy is the recommended treatment approach for primary trigeminal neuralgia (PTN), with interventional surgery procedures considered only when medication therapy proves ineffective or when side effects of medications become intolerabl (3–5).Currently, the most prevalent surgical interventions for PTN include trigeminal microvascular decompression (MVD) and percutaneous balloon compression (PBC) of the trigeminal ganglion. MVD is regarded as the preferred surgical option due to its favorable long-term efficacy (6), while PBC offers advantages such as smaller incisions and a lower surgical risk, particularly in elderly patients with compromised physical conditions (7, 8). But in our clinical experience, we have observed that PBC is generally more acceptable for a wide range of patients, including both elderly individuals but also younger patients in good physical conditions, This acceptability is largely due to PBC's optimal surgical outcomes, minor complications within the acceptable range, shorter operation duration and postoperative hospital stay. Based on the aforementioned advantages, we believe that PBC surgery may be considered as an alternative and potentially the preferred surgical approach for treating PTN, replacing MVD. To support our hypothesis, we conducted a retrospective analysis of 114 cases treated at our center from January 2018 to December 2021 of our center, examining the efficacy, complications, and pain relief rates associated with both procedures. Our goal is identify a more optimal surgical approach for the benefit of patients suffering from PTN.

## 2 Patients and methods

### 2.1 Patients' characteristics

**Figure 1:**
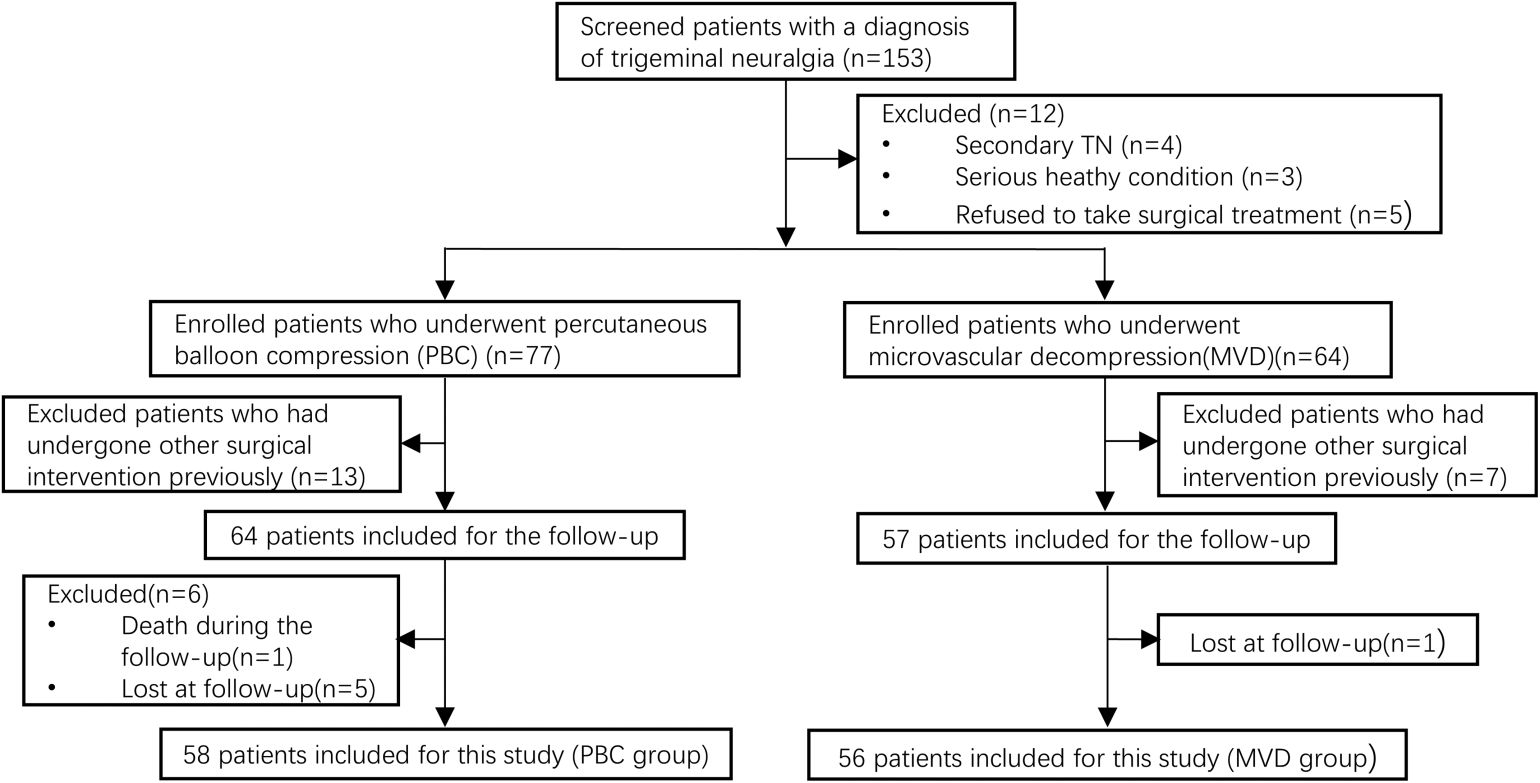
Flow chat of patient selection in this study.

The study was approved by the Ethics Committee of the Gansu Provincial Hospital (2024-610). From January 2018 to December 2021, a total of 158 patients diagnosed with TN and consecutively admitted to the Department of Neurosurgery in Gansu Provincial Hospital, 114 patients were finally included in the study and successfully completed the follow-up after screening. The workflow diagram of these patients was illustrated in Figure 1.

The inclusion criteria were as follows: 1. All patients were diagnosed with PTN based on the third edition of the International Classification of Headache Disorders (ICHD−3), all of them were unilateral pain. 2. Patients with unsatisfactory therapeutic effects of oral medication or who had intolerable adverse drug reactions. 3. The patient and their family have been informed of the surgical risks, have requested surgical treatment and signed the surgical consent form.

The exclusion criteria were as follows: 1. Secondary trigeminal neuralgia. 2. Patient also suffered from other chronic headaches or had a serious condition such as cancer. 3. Patient had previously undergone other surgical intervention for PTN prior to this operation. 4. The patient and their family have declined to participate in the study, or the patient has not cooperated with the follow-up.

### 2.2 Operative procedure

In this study, patients under the age of 70 who presented with satisfactory physical conditions and exhibited visible neurovascular conflict (NVC) on preoperative magnetic resonance imaging (MRI) were recommended for MVD (9). Conversely, patients over the age of 70, those with adverse physical conditions, individuals without distinguishable NVC observable on their preoperative MRI, or those with an extreme apprehension regarding craniotomy underwent PBC.

MVD: After induction of general anesthesia with orotracheal intubation, the patients were placed in the lateral decubitus position with the affected side facing upward. MVD was performed via a standard retromastoid craniotomy. The primary surgical procedure entailed identifying the blood vessel responsible for compressing the trigeminal nerve root and meticulously dissociating it from any adherent vessels. The responsible vessel was insulated from the compressed nerve using a Teflon pledget. Prior to the closure of the skull, a thorough inspection of the entire course of the trigeminal nerve was conducted to confirm that it was free from compression by any vessels.

PBC: All patients who underwent the procedure were positioned supine after general anesthesia with orotracheal intubation. The modified Hartel anterior approach was utilized for puncturing the foramen ovale, with needle insertion guided by C-arm fluoroscopy. The insertion point of entry into the skin was identified approximately 2.5 cm lateral to the corner of the mouth on the affected side. Once the needle reached the foramen ovale successfully, a 14G balloon catheter was inserted into Meckel's cavity, followed by an injection of 0.7–1 ml of Omnipaque water-soluble into the balloon, the position of the catheter was constantly adjusted until the balloon was pear-shaped or pear-like shape. The duration of compression varied between 120 and 150 s. Afterward, the balloon was deflated; both catheter and needle were withdrawn while applying compression at the puncture site for 3 min before concluding the procedure.

### 2.3 Data collection

1. The general characteristics of the patients including age, gender, pain location, side of the facial pain, and disease duration, were assessed. Additionally, data on operation duration including the type of responsible vessel in MVD, balloon shape, contrast agent dosage, and compression duration in PBC; postoperative hospital stay were recorded. Complications observed included facial numbness, masseter muscle weakness, hearing loss, meningitis, cerebrospinal fluid leakage, “ant walking” sensation, herpes simplex, eye dryness, and diplopia. Pain relief was evaluated at three time points: immediate relief (24 h postoperatively), short-term relief (6 months and 1 year postoperatively), long-term relief (2 years postoperatively). Recovery from facial numbness was also monitored.
2. The Barrow Neurological Institute (BNI) Pain Scale was used to evaluate the pain relief after surgery (Table 1), BNI I and BNI II were considered as effective treatment, BNI III, IV, and V indicated poor surgical outcomes or recurrence of pain. Pain relief rate = (Number of patients with BNI I + Number of patients with BNI II)/Total number of patients. The Barrow Neurological Institute (BNI) Numbness Scale was used to evaluate the recovery of facial numbness after surgery (Table 2), BNI I and BNI II were regarded as acceptable degrees of facial numbness, BNI III, IV, and V indicated that facial numbness was bothersome.
3. Data were analyzed using SPSS Statistics 26.0. Quantitative data with a normal distribution were expressed as mean ± standard deviation (x ± s), and group comparisons were performed using the independent samples *t*-test. For quantitative data not following a normal distribution, results were presented as median and interquartile range [*M* (*P*_25_, *P*_75_)], with group comparisons made using the Mann-Whitney U test. Normality assessment was performed using Q-Q plots. Categorical data were presented as frequency and percentage [*n* (%)]. The Chi-square test and Fisher's exact test were used for unordered categorical data, while ordinal categorical data were analyzed using the Mann-Whitney U test. *P* ≤ 0.05 was considered statistically significant.

**Table 1:** The barrow neurological institute (BNI) pain scale.

BNI pain score	Description
I	No pain, not taking any medications
II	Occasional pain, not taking any medications
III	Some pain, adequately controlled with medications
IV	Some pain, not adequately controlled with medications
V	Severe pain or not relief

**Table 2:** The barrow neurological institute (BNI) numbness scale.

BNI numbness score	Description
I	No facial numbness
II	Mild facial numbness that is not bothersome
III	Somewhat bothersome facial numbness
IV	Very bothersome facial numbness

## 3 Results

### 3.1 Patients' characteristics

**Figure 2:**
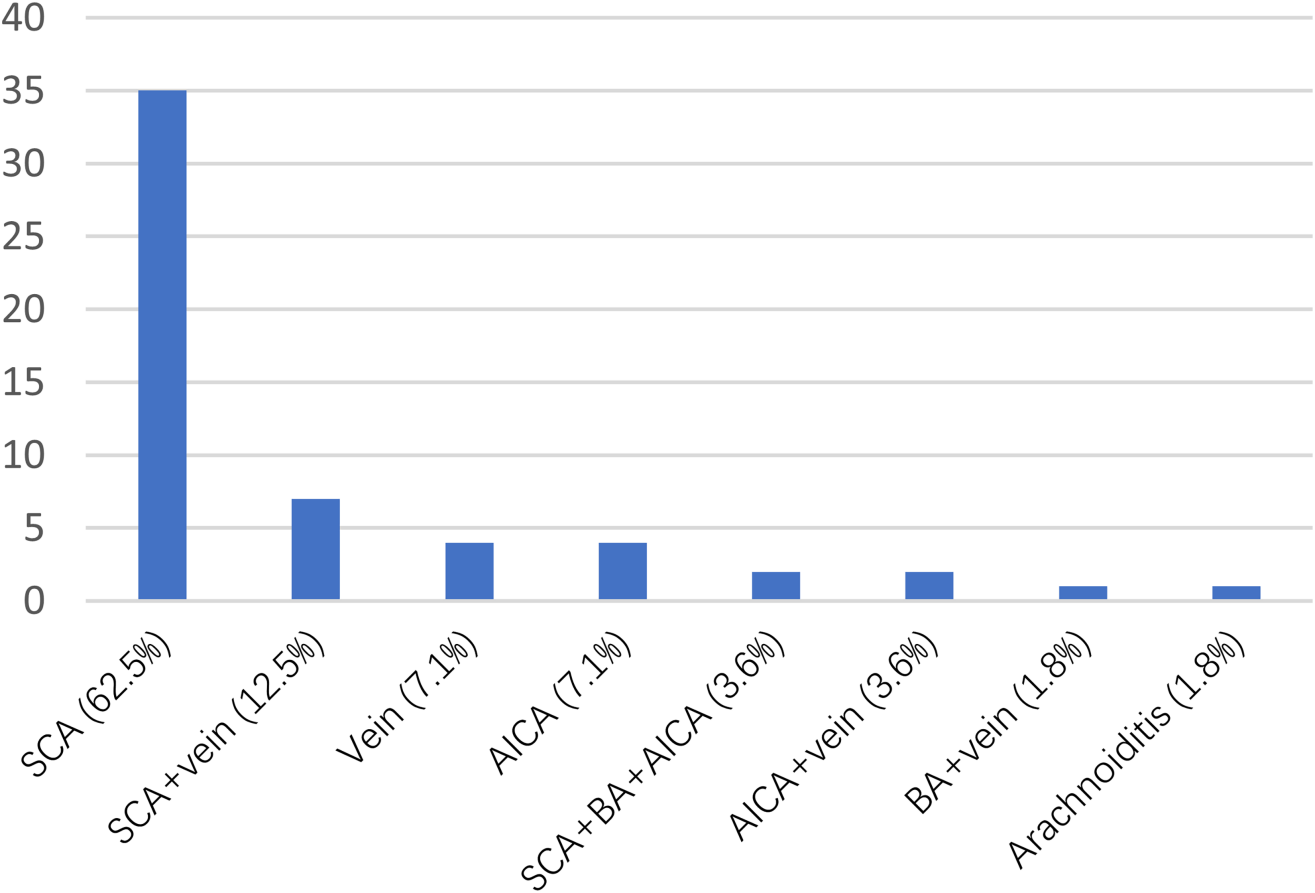
Vessels compressing the trigeminal nerve as identified during operation.

The age of the MVD group (60.18 ± 10.29) was significantly younger than that of the PBC group (70.74 ± 8.59) (*P* < 0.05). Both groups of patients exhibited a higher prevalence of female patient, with MVD group comprising 66.1% and PBC group 67.2%. Pain distribution in both cohorts was mainly V2 and V3 regions, with MVD showing 44.6% and PBC showing 43.1% (Table 3). During MVD exploration, the most common type of responsible vessel was superior cerebellar artery (SCA), accounting for (65%) (Figure 2).

**Table 3:** Comparison of demographic and clinical characteristics between MVD group and PBC group [*n* (%) or *x̄* ± *s* or *M* (*P*_25_, *P*_75_)].

	MVD Group (*n* = 56)	PBC Group (*n* = 58)	*Z*/*χ*^2^/*t* score	*P*
Gender				
Male	19 (33.9)	19 (32.8)	*χ*^2^ = 0.018	0.895
Female	37 (66.1)	39 (67.2)		
Age (years)	60.18 ± 10.29	70.74 ± 8.59	*t* = 5.958	0.000
Course of disease (years)	3.0 (1.0,6.0)	4.0 (2.0,8.0)	*Z* = 1.599	0.110
Side				
L	21 (37.5)	31 (53.4)	*χ*^2^ = 2.921	0.087
R	35 (62.5)	27 (46.6)		
Pain location				
V1	7 (12.5)	0	*χ*^2^ = 16.513	0.006
V2	4 (7.1)	3 (5.2)		
V3	6 (10.7)	2 (3.4)		
V1, V2	9 (16.1)	10 (17.2)		
V2, V3	25 (44.6)	25 (43.1)		
V1, V2, V3	5(8.9)	18(31.0)		

### 3.2 Surgical efficacy

There was no statistically significant difference (*P* = 0.603) in immediate pain relief (24 h postoperatively) between the MVD group (91.07%) and the PBC group (96.55%). No significant differences were found in relief rates at 6 months postoperatively (MVD87.5%, PBC94.8%), 1 year postoperatively (MVD83.90%, PBC94.80%), or 2 years postoperatively (MVD78.60%, PBC72.40%).

### 3.3 Complications and recovery from complications

The incidence of meningitis was notably higher in the MVD group (12.5%) compared to the PBC group (*P* = 0.017), while facial numbness occurred exclusively in all patients from the PBC group (100%), higher than that observed in the MVD group (*P* = 0.000). Among patients experiencing facial numbness after PBC, 10.3% achieved a complete relief (BNI I) at 6 months postoperatively, while only 8.6% exhibited bothersome facial numbness (BNI III) at 2 years postoperatively.

### 3.4 Surgical duration and postoperative hospital stay

Surgical duration and postoperative hospital stay were significantly longer for patients undergoing MVD procedures (3.25 h, 7d) compared to those receiving PBC treatment (0.8 h, 1.5d), with these differences achieving statistical significance (*P* < 0.05).

## 4 Discussion

All surgical procedures in this study were performed by a single surgeon (Dr. Peng). The general characteristics of the patients are summarized in Table 3: both the MVD and PBC groups demonstrated a higher prevalence of female patients (MVD66.1%, PBC67.2%), as well as a greater proportion of pain distribution localized to the V2 and V3 branches of the trigeminal nerve (MVD44.6%, PBC43.1%), consistent with the findings reported in previous studies (10–12). Previous studies have predominantly reported a higher prevalence of facial pain on the right side (10, 12). However, our study did not identify a statistically significant differences in the lateral distribution of facial pain, which may be attributed to the limited sample size.

### 4.1 The surgical outcomes of the two groups were comparable

To differentiate between short-term and long-term relief rates, we considered the immediate relief rate as the effectiveness rate of the surgery. The immediate postoperative pain relief rates (within 24 h) were 91.07% for the MVD group and 96.66% for the PBC group, with no significant statistical difference (*P* = 0.603) (Table 4). This result indicates that there was no significant difference in effectiveness between the two surgical techniques, which is consistent with the findings of other studies (13, 14).

**Table 4:** Comparison of 24 hours postoperatively relief score and relief rates between the MVD group and PBC group [*n* (%)].

Group	*n*	BNI pain score				Relief rates
		BNI1	BNI2	BNI3	BNI4	
MVD	56	48 (85.7)	3 (5.4)	3 (5.4)	2 (3.6)	51 (91.07)
PBC	58	47 (81.0)	9 (15.5)	0 (0.0)	2 (3.4)	56 (96.55)
Z	Z = 0.520					0.686
P	*P* = 0.603					0.407

We believe that the primary reason for inadequate immediate relief following MVD is insufficient decompression of the trigeminal nerve. One possibility is that the responsible vessel is excessively large and tortuous (e.g., the vertebrobasilar artery), and simply using a Teflon pledget to address the neurovascular conflict may not achieve adequate decompression; additional methods, such as vascular suspension, may be necessary. Another possibility is the presence of stenosis in the bony structures through which the trigeminal nerve exits the skull, leading to compression of the nerve by the accompanying vessels. Only two patients experienced poor relief after PBC. We consider that the primary reasons for the inadequate immediate relief following PBC may be insufficient amounts of contrast agent and inadequate compression duration.

In a study by Giorgio Cruccu et al., the relief rate following MVD was reported to be 68%-88% within 1–2 years, decreasing to 61%–80% at the 4–5 years postoperatively. The average relief rate for PBC over a period of 4.2–7 years postoperatively was found to be 68%, whereas radiofrequency thermocoagulation had an average relief rate of 58% over a follow-up of 3–9.3 years postoperatively, the average relief rate for glycerol rhizolysis across a follow-up of 4.5–8 years was only 28% (15). However, our study results indicate that there is no significant difference in the short-term and long-term relief rates following MVD and PBC surgeries. Only the data from 2 years postoperatively showed that the relief rate for MVD (78.6%) was slightly higher than that for PBC (72.4%), but this difference was not statistically significant (*P* = 0.08). (Table 5).

**Table 5:** Comparison of pain score between MVD group and PBC group at various time points [*n* (%)].

Group	*n*	6 months postoperatively					1 year postoperatively					2 years postoperatively				
		BNI1	BNI2	BNI3	BNI4	BNI5	BNI1	BNI2	BNI3	BNI4	BNI5	BNI1	BNI2	BNI3	BNI4	BNI5
MVD	56	45 (80.4)	4 (7.1)	4 (7.1)	2 (3.6)	1 (1.8)	43 (76.8)	4 (7.1)	3 (5.4)	4 (7.1)	2 (3.6)	41 (73.2)	3 (5.4)	2 (3.6)	7 (12.5)	3 (5.4)
PBC	58	42 (72.4)	13 (22.4)	1 (1.7)	1 (1.7)	1 (1.7)	42 (72.4)	13 (22.4)	1 (1.7)	1 (1.7)	1 (1.7)	33 (56.9)	9 (15.5)	1 (1.7)	7 (12.1)	8 (13.8)
*Z*				0.706					0.145					1.750		
*P*				0.480					0.885					0.080		

In a prospective study conducted by Tone Bruvik Heinskou et al. in 2019, it was found that gender and the severity of NVC were significantly correlated with the prognosis of MVD. Specifically, male patients and those with more severe NVC exhibited better surgical outcomes (16). However, this finding is controversial, as other studies suggest that prognosis may be influenced by various factors such as age, disease duration, the present of trigger points and paroxysmal pain (17–19). Currently, there is no consensus on the factors affecting MVD surgical outcomes. In our study, during the two-year follow-up in the MVD group, aside from the 5 patients with poor immediate surgical outcomes, a total of 5 patients experienced recurrence within the 2 years postoperatively. Among these 5 patients, there were 3 females and 2 males, with ages ranging from 38 to 69 years and disease durations varying from 1 to 10 years. The pain distribution areas included V2 + V3 for four patients and V1 + V2 + V3 for one patient. The types of responsible vessels identified during surgery were as follows: four cases involving the SCA and one case involving the anterior inferior cerebellar artery (AICA). We conducted a preliminary analysis of these characteristics and found no significant correlation between recurrence and the aforementioned features. Unfortunately, we were unable to record and classify the degree of vascular compression shown on preoperative MRIs; thus, our study cannot establish a relationship between the degree of vascular compression and disease recurrence. Furthermore, regarding trigger points and paroxysmal pain, nearly all patients had identifiable trigger points, while persistent pain was rare; therefore, this study also cannot determine whether these factors influence prognosis. We believe that three potential reasons may account for short-term or long-term recurrence: first, adhesions may develop at the surgical site due to chronic inflammation; second, the Teflon pledget or responsible vessel may have shifted, leading to renewed nerve compression; and third, the Teflon pledget may form inflammatory granulomas, resulting in new compression of the nerve.

Currently, we have not found any reports analyzing factors related to the prognosis of PBC. There is only one report on the short-term relief rate of PBC, which suggests that the short-term relief rate may be associated with the response to carbamazepine and the degree of vascular compression on the nerve observed in preoperative MRI (20). In our study, we attempted a preliminary analysis of cases of ineffective outcomes and recurrence following PBC surgery, but the results were similarly disappointing to those in the MVD group, as we did not identify any factors that might impact the prognosis of PBC. We believe that the success of PBC cannot be solely determined by the recurrence rate; it is essential to seek a balance between surgical outcomes and postoperative complications (such as facial numbness, sensory reduction, and decreased corneal reflex). Recurrence does not necessarily equate to failure. If complete pain relief is to be absolutely guaranteed, patients may have to endure intolerable postoperative complications.

In this study, the immediate, short-term, and long-term relief rates for MVD were not statistically significantly different from those of PBC, which contrasts slightly with previous studies. This discrepancy may be attributed to factors such as a small sample size and insufficient follow-up duration. Nevertheless, our center posits that while the long-term relief rate for PBC may not as favorable as that of MVD, there are currently numerous surgical options available for treating PTN, allowing patients to select various approaches to deal with the recurrence of pain after the initial surgical intervention. Percutaneous destructive neurosurgical techniques - such as radiofrequency thermocoagulation, glycerol rhizolysis, and balloon compression - are relatively straightforward and demonstrate a definitive therapeutic effect (21, 22). Additionally, PBC is generally more acceptable to patients, both physically and psychologically, compared to invasive craniotomy.

### 4.2 The PBC group had fewer severe complications compared to the MVD group

With regard to postoperative complications (Table 6), the incidence of meningitis in the MVD group was 12.5%, significantly higher than that observed in the PBC group (*P* < 0.05). However, all cases of meningitis were mild and resolved successfully with treatment, with no long-term complications reported. It is noteworthy that the incidence of meningitis reported in previous studies ranged from 0% to 2% (6, 14, 23), which was considerably lower than the findings in this study. Bacterial smear and culture examinations were conducted for every patient with meningitis; however, the results were negative in almost all cases. This was primarily due to the low positive rate of bacterial smear and culture in cerebrospinal fluid (CSF), even in confirmed cases of bacterial meningitis, where CSF culture results could still be negative. Consequently, it was not possible to definitively determine whether the meningitis was bacterial or aseptic. Instead, an indirect assessment was made based on the sugar content of CSF obtained through biochemical examination, however, the sugar content in CSF could be influenced by the patient's blood glucose levels at the time of collection. We hypothesized that postoperative meningitis was often caused by inadequate aseptic techniques during surgery. To mitigate this, we enhanced the type of disinfectant used for preoperative preparation, implemented prophylactic antibiotics prior to surgery, and imposed stricter aseptic protocols for all personnel involved in the surgical procedures.

**Table 6:** Comparison of complications between the MVD group and PBC group [*n* (%)].

	MVD group (*n* = 56)	PBC group (*n* = 58)	*χ* ^2^	*P*
Meningitis	7 (12.5)	0	5.708	0.017*
Cerebrospinal fluid leakage	1 (1.8)	0	—	0.491#
Ant walking sensation	0	3 (5.2)	1.299	0.254*
Masseter muscle weakness	0	2 (3.4)	—	0.496
Hearing loss	4 (7.1)	0	2.443	0.118
Facial numbness	4 (7.1)	58 (100.0)	99.028	0.000
Eye dryness	2 (3.6)	3 (5.2)	0.000	1.000
Herpes simplex	0	0	—	—
Diplopia	4 (7.1)	0	2.443	0.118

* Indicates the continuity-corrected chi-square test.

^#^ Indicates the Fisher's exact probability method.

The incidence of facial numbness (BNI > I) following PBC was 100%, making it the only significantly high-incidence complication compared to MVD. Facial numbness arises from the lesions at the large myelinated fibers; however, the therapeutic efficacy of PBC is also attributed to this mechanical damage by ballon inflation (10, 24). Consequently, it can be regarded as an unavoidable complication of PBC. The effectiveness of PBC and the duration of postoperative facial numbness are related with many factors such as balloon pressure, compression time, and balloon shape during the procedure (25, 26). In this study, based on our experience, we kept the contrast agent dosage between 0.7 and 1 ml, maintaining the balloon shape as pear or pear-like, and controlled the compression time to between 120 and 150 s. We consider this approach to be the optimal choice for balancing surgical effectiveness and postoperative complications. Despite the relatively high incidence of facial numbness following PBC, this symptom was generally considered acceptable for patients experiencing long-term pain. During follow-up assessments, patients classified as BNI II reported no significant impact on their daily life. Moreover, 10.3% of the patients with facial numbness experienced complete relief (BNI I) at 6 months postoperatively, while only 8.6% exhibited bothersome facial numbness (BNI III) at 2 years postoperatively. Notably, none of the 58 PBC patients in this study experienced severe facial numbness (BNI IV) that adversely affected their quality of life (Table 7).

**Table 7:** Comparison of numbness score between MVD group and PBC group at various time points [*n* (%)].

Group	*n*	6 months postoperatively			1 year postoperatively			2 years postoperatively		
		BNI1	BNI2	BNI3	BNI1	BNI2	BNI3	BNI1	BNI2	BNI3
MVD	56	52 (92.9)	3 (5.4)	1 (1.8)	52 (92.9)	4 (7.1)	0	53 (94.6)	3 (5.4)	0
PBC	58	6 (10.3)	46 (79.3)	6 (10.3)	6 (10.3)	46 (79.3)	6 (10.3)	20 (34.5)	33 (56.9)	5 (8.6)
Z		8.463			8.656			6.642		
P		0.000			0.000			0.000		

Another significant postoperative complication is hearing loss, which, despite its low incidence (1.8%) (27), can exert long-term serious adverse effects on patients' daily life. In this study, 4 cases (7.1%) of hearing loss were identified in MVD group during follow-up assessments. We believe there are two main causes of hearing loss: excessive traction of the auditory nerve during surgery and damage to the blood vessels supplying the auditory nerve. The prognosis for hearing loss following MVD was found to be poor, all 4 patients underwent various treatments for their condition, including hyperbaric oxygen, over a two-year follow-up period, with minimal efficacy observed. This situation imposed considerable psychological and physical stress on the patients. Notably, we did not encounter any patients who developed neurotrophic corneal ulcers postoperatively.

### 4.3 The surgical time and postoperative hospitalization duration in the PBC group were shorter than those in the MVD group

This study documented the surgical duration and postoperative hospital stay for both procedures (Table 8). The findings indicate that the average surgical duration for MVD was 3.25 h, significantly longer than the average of 0.8 h observed for PBC. A shorter surgical duration is associated with a reduced risk related to general anesthesia (28). Furthermore, patients undergoing PBC had an average postoperative hospital stay of 1.5 days, which was more favorable compared to the 7 days required following MVD.

**Table 8:** Comparison of operative duration, hospital duration postoperatively between MVD group and PBC group [*M* (*P*_25_, *P*_75_)].

	MVD group (*n* = 56)	PBC group(*n* = 58)	*Z*	*P*
Operative duration (h)	3.25 (2.93, 3.74)	0.8 (0.5, 1.0)	*Z* = 9.207	0.000
Hospital duration postoperatively (days)	7.0 (5.0, 9.75)	1.5 (1.0, 2.0)	*Z* = 7.906	0.000

## 5 Limitation

This study is retrospective in nature and is subject to the inherent limitations of such designs, including incomplete data, insufficient follow-up duration, and a lack of randomization in case selection, which may introduce selection bias. These factors could affect the evaluation of surgical efficacy. In the future, our center may consider conducting a prospective study to ensure a more reliable assessment of surgical outcomes.

## 6 Conclusion

This retrospective study indicates that MVD and PBC exhibit nearly identical immediate, short-term, and long-term relief rates postoperatively. Additionally, PBC is characterized by its simplicity, shorter operative time, reduced risks associated with general anesthesia, fewer complications, and a shorter hospital stay postoperatively. Based on the aforementioned reasons, our center believes that PBC may serve as a viable alternative to MVD as the preferred surgical approach for treating PTN in the future.

## Data Availability

The original contributions presented in the study are included in the article/Supplementary Material, further inquiries can be directed to the corresponding authors.
